# Aerobic Continuous and Interval Training under Hypoxia Enhances Endurance Exercise Performance with Hemodynamic and Autonomic Nervous System Function in Amateur Male Swimmers

**DOI:** 10.3390/ijerph18083944

**Published:** 2021-04-09

**Authors:** Sung-Woo Kim, Won-Sang Jung, Jeong-Weon Kim, Sang-Seok Nam, Hun-Young Park

**Affiliations:** 1 Physical Activity and Performance Institute, Konkuk University, 120 Neungdong-ro, Gwangjin-gu, Seoul 05029, Korea; kswrha@konkuk.ac.kr (S.-W.K.); jws1197@konkuk.ac.kr (W.-S.J.); 2 Graduate School of Professional Therapy, Gachon University, 1332 Seongnam-daero, Sujeong-gu, Seongnam-si 13306, Korea; zeezone@gachon.ac.kr; 3 Taekwondo Research Institute of Kukkiwon, 32 Teheran7gil, Gangnam-gu, Seoul 06130, Korea; play_data@naver.com; 4 Department of Sports Medicine and Science, Graduate School, Konkuk University, 120 Neungdong-ro, Gwangjin-gu, Seoul 05029, Korea

**Keywords:** aerobic continuous and interval training, amateur male swimmers, autonomic nervous system function, endurance exercise performance, hemodynamic function, hypoxia

## Abstract

Hypoxic training is often performed by competitive swimmers to enhance their performance in normoxia. However, the beneficial effects of aerobic continuous and interval training under hypoxia on hemodynamic function, autonomic nervous system (ANS) function, and endurance exercise performance remain controversial. Here we investigated whether six weeks of aerobic continuous and interval training under hypoxia can improve hematological parameters, hemodynamic function, ANS function, and endurance exercise performance versus normoxia in amateur male swimmers. Twenty amateur male swimmers were equally assigned to the hypoxic training group or normoxic training group and evaluated before and after six weeks of training. Aerobic continuous and interval training in the hypoxia showed a more significantly improved hemodynamic function (heart rate, −653.4 vs. −353.7 beats/30 min; oxygen uptake, −62.45 vs. −16.22 mL/kg/30 min; stroke volume index, 197.66 vs. 52.32 mL/30 min) during submaximal exercise, ANS function (root mean square of successive differences, 10.15 vs. 3.32 ms; total power, 0.72 vs. 0.20 ms^2^; low-frequency/high-frequency ratio, −0.173 vs. 0.054), and endurance exercise performance (maximal oxygen uptake, 5.57 vs. 2.26 mL/kg/min; 400-m time trial record, −20.41 vs. −7.91 s) than in the normoxia. These indicate that hypoxic training composed of aerobic continuous and interval exercise improves the endurance exercise performance of amateur male swimmers with better hemodynamic function and ANS function.

## 1. Introduction

Endurance exercise performance is highly correlated with various physiological components that can be altered by diversiform training methods under hypoxia, including hematological changes such as erythropoiesis and nonhematological changes such as exercise economy, hemodynamic function, capillary density, and acid-base response in the skeletal muscle [1,2]. Accordingly, exercise training under hypoxia has been used worldwide for decades to enhance endurance exercise performance [3,4,5].

Hypoxic training can reinforce endurance exercise performance by using three training methods. Living high training high (LHTH) was the first hypoxic training design to living and training design at 1500–4000 m in the natural altitude conditions that improve hematological function, including erythropoiesis and oxygen delivery capacity [6,7]. However, LHTH has a major limitation in that it fails to achieve training of the same intensities (e.g., exercise speed and load) as training in a normoxic condition [8]. To overcome these shortcomings of LHTH, living high training low (LHTL), living high at 2000–3000 m and simultaneously training low at below 1500 m, was developed by Dr. Benjamin Levine and James Stray-Gundersen of the United States in the early 1990s [8]. LHTL simultaneously offers athletes the beneficial effects of hematological function (e.g., improved erythropoiesis) and normoxic training (i.e., maintenance of training intensity) [8,9]. In recent years, living low training high (LLTH) has received the most attention from various athletes because it is generally associated with shorter exposure time to hypoxia (approximately three to five sessions per week of 1–3 h), less effort, less time, and lower cost than LHTH and LHTL [1,10].

LLTH for exercise performance consists of various types of training methods such as continuous hypoxic training (CHT), interval hypoxic training (IHT), sprint interval training under hypoxia (SIH), repeated sprint training under hypoxia (RSH), resistance training under hypoxia (RTH), and voluntary hypoventilation at a low lung volume (VHL) [11]. Among these various LLTH methods (e.g., CHT, IHT, SIH, RSH, RTH, and VHL), CHT and IHT are most commonly used to enhance endurance exercise performance [1,4,5,10]. However, few studies have examined the effects of hypoxic training consisting of CHT and IHT on endurance exercise performance (especially swimming performance) versus continuous and interval training in normoxia. Short-term exposure to hypoxia with high-intensity exercise such as CHT, IHT, and CHT + IHT improves endurance exercise performance by enhancing metabolic function, hemodynamic function, and exercise economy [1,5,10,12,13]. However, some studies reported that CHT, IHT, and CHT + IHT did not enhance endurance exercise performance [14,15,16,17]. These conflicting results are because changes in autonomic nervous system (ANS) function, which are highly correlated with endurance exercise performance, have not been reviewed [10,18,19,20].

Heart rate variability (HRV) reflects the interaction between the sympathetic nervous system (SNS) and parasympathetic nervous system (PNS), which regulate cardiovascular function, and is an effective test method for quantitatively evaluating ANS activity and balance [21]. Dynamic modulation of cardiac and peripheral vascular regulation, including their regulation by the ANS, occurs in response to rapid changes in heart rate (HR) [21,22]. The improvement of HRV function via exercise training is often interpreted as enhanced ANS balance function or vagus nerve activity, which is related to endurance exercise performance [23,24]. Therefore, it is essential to verify the effect of exercise training under hypoxia on changes in HRV related to endurance exercise performance versus normoxia to verify the effectiveness of hypoxic training. However, studies to date of changes in ANS function following hypoxic training are scarce.

Therefore, the purpose of this study was to evaluate the effects of aerobic continuous and interval training under hypoxia on hemodynamic function, ANS function, and endurance exercise performance in amateur male swimmers. We hypothesized that aerobic continuous and interval training under hypoxia would improve endurance exercise performance by enhancing hemodynamic and ANS function in amateur male swimmers versus normoxia.

## 2. Materials and Methods

### 2.1. Subjects

The subjects were competitive amateur male swimmers (*n* = 20) with no experience in any exercise and training program in normobaric or hypobaric hypoxia (Table 1). They were equally assigned to the normoxic training group (NTG; *n* = 10) or hypoxic training group (HTG; *n* = 10) according to endurance exercise performance and body composition. We explained the experiments and possible side effects to all amateur male swimmers prior to the start of the study and obtained signed consent for participation. The present study was approved by the Institutional Review Board of Konkuk University (7001355-201510-HR-090) in Korea and was conducted in accordance with the provisions of the Declaration of Helsinki.

### 2.2. Study Design

**Figure 1:**
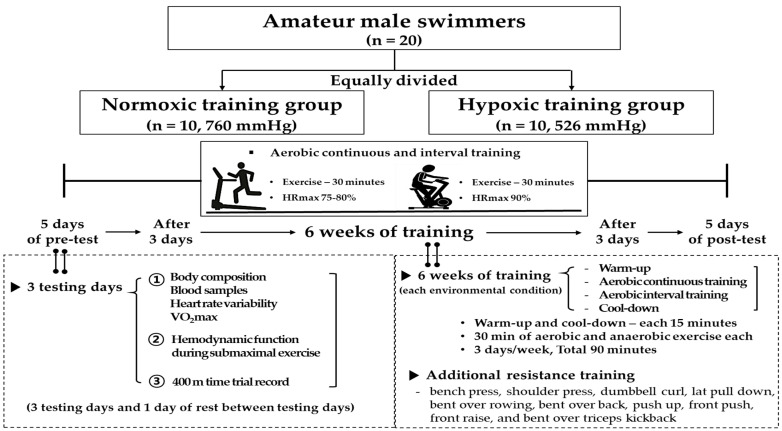
Study design. HR_max_, maximal heart rate; VO_2max_, maximal oxygen uptake.

The present study design is illustrated in Figure 1. Twenty amateur male swimmers were equally divided into the NTG (*n* = 10; aerobic continuous and interval training in a normoxia; 760 mmHg) and HTG (*n* = 10; aerobic continuous and interval training in a hypobaric hypoxia; 526 mmHg; simulated altitude of 3000 m). All testing and training sessions were performed in a 6.5 m wide × 7.5 m long × 3 m high hypobaric hypoxic chamber (Submersible Systems, Huntington Beach, CA, USA). The temperature within the hypobaric hypoxic chamber was maintained at 20 ± 2 °C, and the humidity was maintained at 60 ± 2%.

The experimental design consisted of the following: a 5-day pre-test period (i.e., 3 testing days and 1 rest day between them), a 6-week training period under each environmental condition, and a 5-day post-test period. The post-test period began 3 days after the final training session. During the 3 days of test sessions, body composition, hematological parameters, hemodynamic function, ANS function, and endurance exercise performance were evaluated.

On the first pre- and post-testing days, venous blood samples were collected between 8:00 and 10:00 a.m. after 12 h of fasting to analyze hematological parameters and serum cortisol levels. Thereafter, the body composition and HRV parameters were measured. Subsequently, the maximal oxygen uptake (VO_2max_) was measured to evaluate the endurance exercise performance in the afternoon. On the second pre- and post-testing days, hemodynamic function parameters were measured during a 30-min bout of submaximal cycle ergometer exercise. The exercise intensity was set at individual cycle ergometer exercise load values corresponding to 70% maximal HR (HR_max_) obtained during the pre-test period. On the third testing day, a 400-m time trial record in freestyle was measured by an automatic system installed on an authorized indoor swimming pool (50 m) at sea level in Suwon.

During the 6-week training period, 20 amateur male swimmers were equally divided into the NTG (*n* = 10) and HTG (*n* = 10), and they performed four kinds of training sessions in each environmental condition (NTG: normoxia, 760 mmHg; HTG: simulated 3000 m hypobaric hypoxia, 526 mmHg) for 90 min: warm-up, aerobic continuous exercise, aerobic interval exercise, and cool-down. The training frequency was 90 min 3 days per week for 6 weeks. Warm-up and cool-down were set at 50% maximum heart rate (HR_max_) for each subject for 5 min, then increased by 10% HR_max_ every 5 min and performed for 15 min. Continuous aerobic exercise was performed on a treadmill (Precor 932i, Precor, WA, USA) at 75% HR_max_ for 30 min and aerobic interval exercise on a cycle ergometer (Monark Exercise AB, Vansbro, Sweden) set at the exercise load with 90% HR_max_ measured in pre-test for 30 min (10 × 2-min exercise and 1-min rest). The velocity during warm-up, aerobic continuous exercise, and cool-down on a treadmill was changed using an HR monitor (Polar S610i, Helsinki, Finland) to match each HR. Anaerobic interval exercise intensity was set at individual bicycle exercise load values (watts) with 90% HR_max_ obtained at pre-test in each environmental condition.

In addition, all participants performed equally additional resistance training sessions (three sets of 8–10 repetitions at an exercise intensity range of 70–80% of one-repetition maximum, with 60-s rest per set) in a normoxia composed of a bench press, shoulder press, dumbbell curl, lat pull-down, bent-over-rowing, bent-over-back, push-up, front push, front raise, and bent-over tricep kickback. Additional resistance training frequency was 60 min 3 days per week for 6 weeks.

All exercise training sessions in normoxia or hypoxia were supervised by researchers, coaches, and directors.

### 2.3. Body Composition

Body composition parameters (e.g., weight, body mass index, and % body fat) were evaluated after 12 h of fasting using bioelectrical impedance analysis. All participants wore lightweight clothing and were asked to remove any metal items. An Inbody 770 device (Inbody, Seoul, Korea) was used to measure body composition.

### 2.4. Hematological Parameters

Venous blood samples were performed on pre- and post-testing days at rest in normoxia to measure hematological parameters. A 5-mL sample of venous blood samples was collected in a heparin tube for whole blood (3-mL) and a serum separation tube for serum (2-mL).

To analyze hematological parameters, blood samples were obtained before and after training at rest at normoxia. A 5-mL sample of venous blood was collected in a heparin tube for whole blood (3-mL) and a serum separation tube for serum (2-mL). An XE2100D hematology analyzer (Sysmex, Kobe, Japan) was used to analyze the red blood cell (RBC) count, hemoglobin (Hb) concentration, and hematocrit (Hct). The RBC count and Hct were measured using an impedance-based method. Hb concentration was measured using cyanide-free Hb spectrophotometry. Erythropoietin (EPO) levels were measured using an Immulite 2000 XPI analyzer (Siemens, Eschborn, Germany) using the chemiluminescent immunoassay method. Mean corpuscular volume (MCV), mean corpuscular hemoglobin (MCH), and mean corpuscular hemoglobin concentration (MCHC) were calculated using the following formulas: MCV = (Hct/erythrocyte) × 10; MCH = (Hb/erythrocyte) × 10; and MCHC = (Hb/Hct) × 100.

### 2.5. Hemodynamic Function Parameters

Hemodynamic function parameters were evaluated on pre- and post-testing days. All subjects performed a submaximal exercise using a cycle ergometer with a load corresponding to 75% HRmax measured before training in normoxia. Oxygen uptake (VO_2_) was measured using a Vmax-229 breath-by-breath auto metabolism analyzer (SensorMedics, Yorba Linda, CA, USA). HR, stroke volume index (SVi), and cardiac output index (COi) were evaluated noninvasively using a thoracic bioelectrical impedance device (PhysioFlow PF-05, Paris, France). All variables were measured every minute, and the total values were used for HR, VO_2_, SVi, and COi.

### 2.6. ANS Function

ANS function was assessed by measuring HRV and serum cortisol levels. After approximately 10 min of rest, four pads were placed on the wrists and ankles using an HRV meter (LAXTHA; CANS-3000, Daejeon, Korea), and all male amateur swimmers’ HRV was evaluated in the resting condition. The following parameters were measured: standard deviation of successive differences (SDNN) and root mean square of successive differences (RMSSD) for the time domain methods and total power (TP), low-frequency (LF), high-frequency (HF), and LH/HF ratio for the frequency domain methods. Serum cortisol levels were determined using radioimmunoassay (Coat-A-count; Siemens, Eschborn, Germany).

### 2.7. Endurance Exercise Performance

To evaluate exercise performance, VO_2max_ was measured before and after training using the BRUCE protocol for graded exercise testing on a treadmill (Precor 932i) with a Vmax-229 breath-by-breath auto metabolism analyzer (SensorMedics) under normoxia. The 400-m time trial records in freestyle were measured twice by an automatic system installed on an authorized indoor swimming pool (50 m) at sea level in Suwon, and the average time was used.

### 2.8. Statistical Analysis

All statistical analyses were conducted using SPSS version 25.0 (IBM Corp., Armonk, NY, USA) for Windows. Data are presented as mean ± standard deviation. The normality of the distribution of all outcome variables was verified using the Shapiro–Wilk test. A two-way analysis (time × group) of variance with repeated measures of the “time” factor was used to analyze the effects of training programs on each dependent variable. Partial eta-squared (η^2^) values were calculated as measures of the effect size. If a significant interaction effect was found, a Bonferroni post hoc test was used to identify intragroup changes over time. Additionally, the paired t-test was used to compare the pre- and post-training values of dependent variables in each group separately. An a priori power analysis was performed with G-power for the hemodynamic function parameter (VO_2_ during submaximal exercise) based on previous research [1], indicating that a sample size of 16 participants (8 participants per group) would be required to provide 88% power at an α-level of 0.05. We anticipated a more than 10% dropout rate and aimed for a starting population of 20. The level of significance was set a priori at *p* < 0.05.

## 3. Results

### 3.1. Body Composition

Table 2 shows the body composition parameters before and after training in both groups. No significant interaction was observed in any of the body composition parameters, that is, body composition did not affect changes in the other dependent parameters.

### 3.2. Hematological Parameters

As shown in Table 3, there was a significant interaction between RBC (*η^2^* = 0.417, *p* = 0.002), Hb (*η^2^* = 0.397, *p* = 0.003), and Hct (*η^2^* = 0.479, *p* = 0.001). Post hoc analyses showed that both groups showed a significant decrease in RBC (HTG −0.32, 95% CI −0.39, −0.24; NTG −0.14, 95% CI −0.22, −0.05 106/uL, *p* < 0.05) and Hb (HTG −0.87, 95% CI −1.11, −0.63; NTG −0.38, 95% CI −0.60, −0.15 kg, *p* < 0.05), but HTG showed a greater decrease than NTG. Hct (HTG −2.41, 95% CI −3.12, −1.70 kg, *p* < 0.05) presented a significant decrease only in HTG. No significant interaction was observed with EPO, MCV, MCH, and MCHC.

### 3.3. Hemodynamic Function

As shown in Table 4, there was a significant interaction between HR (*η^2^* = 0.256, *p* = 0.023), VO_2_ (*η^2^* = 0.237, *p* = 0.030), and SVi (*η^2^* = 0.308, *p* = 0.011). Post hoc analyses found significant decrease in both groups for HR (HTG, −653.4; 95% CI, −795.4 to −511.4 beats/30 min; NTG, −353.7; 95% CI, −586.1 to −121.3 beats/30 min; *p* < 0.05) and SVi (HTG, 197.66; 95% CI, 90.10–305.22 mL/30 min; NTG, 52.32; 95% CI, 8.54–96.10 mL/30 min; *p* < 0.05). VO_2_ (HTG, −62.45; 95% CI, −91.16 to −33.74 mL/kg/30 min; *p* < 0.05) showed a significant decrease in the HTG only. However, no significant interaction was observed for COi. Overall, hemodynamic function improved more in the HTG than in the NTG.

### 3.4. ANS Function

Table 5 presents a significant interaction in RMSSD (*η^2^* = 0.227, *p* = 0.034), TP (*η^2^* = 0.405, *p* = 0.003), and LF/HF ratio (*η^2^* = 0.226, *p* = 0.034). Post hoc analyses revealed that the HTG had a significantly improved mean RMSSD (HTG, 10.15; 95% CI, 4.37–15.94 ms^2^; *p* < 0.05), TP (HTG, 0.72; 95% CI, 0.50–0.94 ms^2^; *p* < 0.05), and LF/HF ratio (HTG, −0.173; 95% CI, −0.277 to −0.069; *p* < 0.05). However, no significant interaction was observed among SDNN, LF, HF, and serum cortisol levels.

### 3.5. Endurance Exercise Performance

**Figure 2:**
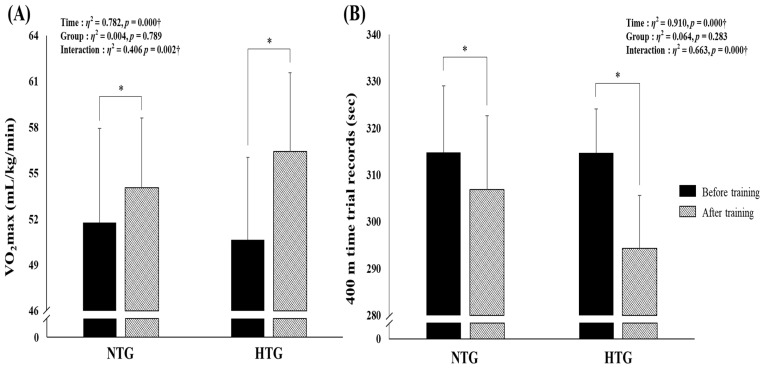
Changes in endurance exercise performance parameters before versus after training by group. (**A**) Change in VO_2max_ before versus after exercise training in each environmental condition. (**B**) Change in the 400-m time trial records before versus after exercise training in each environmental condition. HTG, hypoxic training group; NTG, normoxic training group; VO_2max_, maximum oxygen uptake. Significant interaction or main effect: † *p* < 0.05; Significant difference before versus after training: * *p* < 0.05.

Figure 2 depicts the body composition parameters before and after training by group. There was a significant interaction between VO_2max_ (*η^2^* = 0.406, *p* = 0.002) and a 400-m time trial record (*η^2^* = 0.663, *p* < 0.001). Post hoc analyses revealed a significantly improved VO_2max_ (HTG, 5.57; 95% CI, 4.19–7.35; NTG, 2.26; 95% CI, 0.64–3.88 mL/kg/min; *p* < 0.05) and 400-m time trial record (HTG, −20.41; 95% CI, −23.68 to −17.14; NTG, −7.91; 95% CI, −11.36 to −4.46 s; *p* < 0.05) in both groups. Consequently, endurance exercise performance induced greater improvement in the HTG than in the NTG.

## 4. Discussion

In our study, we hypothesized that aerobic continuous and interval training under hypoxia (simulated 3000 m, 526 mmHg hypobaric hypoxia) would improve endurance exercise performance with enhanced hemodynamic and ANS function more than under normoxia in amateur male swimmers. The present study aimed to demonstrate that our hypoxic training program can sufficiently elicit a meaningful increase in endurance exercise performance with enhanced hemodynamic and ANS function in amateur male swimmers. Our findings are consistent with these hypotheses.

In general, exposure to hypoxia reportedly stimulates erythropoiesis to enhance the oxygen delivery capacity in people living under normoxia [25,26]. Erythropoiesis caused by hypoxia involves an increase in the mass of Hb, a metal protein that carries oxygen in the RBC, and an increased RBC count [27]. In addition, EPO has the greatest influence on hematological changes caused by hypoxia [9]. When exposed to hypoxia, the EPO concentration acutely increases and peaks at approximately 48–72 h, and the production of new by EPO stimulation reportedly takes nearly five days [25,26,28]. An increase in erythropoiesis and a decrease in plasma volume due to exposure to hypoxia means that the ability to deliver oxygen per unit of blood increases, but a decrease in plasma volume can adversely affect the oxygen delivery capacity through the circulatory system. In practice, endurance exercise performance in athletes is largely determined by the decrease in Hct and blood viscosity according to plasma volume [9,29]. However, exercise training under hypoxia such as LHTH and LHTL is very effective at improving blood oxygen delivery capacity because it induces an increase in plasma volume as well as an increase in RBC production via EPO, and these effects were maintained for approximately 16 days after exposure to hypoxia [26,29,30].

The aerobic continuous and interval training under hypoxia used in our study is an LLTH (residing in normoxia but training under hypoxia), which does not induce an increase in hematological changes due to insufficient hypoxia stimulation because the exposure time to hypoxia is substantially less than 2 h per day [29,31]. In our study, six weeks of aerobic continuous and interval training under hypoxia did not show increased erythropoiesis, examined via hematological changes compared with normoxia. RBC count (HTG, −0.32 × 10^6^/µL vs. NTG −0.14 × 10^6^/µL) and Hb concentration (HTG, −0.87 g/dL vs. NTG, −0.38 g/dL) decreased significantly in both groups, and the reduction was greater in the HTG than in the NTG. However, Hct showed a significant decrease only in the HTG (−2.41%). In other words, the greater decrease in RBC count and Hb concentration presented in the NTG is thought to be due to a greater increase in plasma volume by hypoxic versus normoxic training. Aerobic continuous and interval training under hypoxia confirmed the possibility of improving the oxygen delivery capacity by inducing a decrease in Hct via increasing plasma volume.

Hemodynamic function refers to the dynamics of blood flow in systemic conditions, and the hemodynamic system plays a role in distributing blood flow via continuous monitoring and adjustment of the conditions in the body and its environment [10]. In particular, hemodynamic function during submaximal exercise is closely related to endurance exercise performance as an indicator of oxygen delivery and use capacity [1,10,12].

Hypoxic training such as CHT, IHT, and CHT + IHT effectively improved endurance exercise performance via increasing glycolysis enzyme activity, glucose delivery capacity, mitochondrial density, capillary density, acid-base balance regulation, cross-sectional area of skeletal muscle, and activity of the motor unit by stimulating the neuromuscular system [1,5,13,29]. These physiological, biochemical, and structural adaptative changes improve the efficiency of oxygen blood delivery and use capacity, thereby enhancing endurance exercise performance [1,5,10,13,32]. In addition, endurance exercise performance is improved by enhancing the exercise economy (defined as the amount of energy per unit distance), which represents the rate of oxygen flow into the skeletal muscle tissue and the ability of mitochondria to utilize oxygen [1,9,33]. Previous studies reported that a greater exercise economy is related to improved endurance exercise performance by physiological adaptations to hypoxic training [1,9,10,34]. However, there is a lack of previous studies that prove that hypoxic training such as CHT, IHT, and CHT + IHT improves endurance exercise performance based on hemodynamic function, which indicates oxygen delivery and utilization capacity in systemic conditions, including exercise economy. Therefore, we verified the effects of aerobic continuous and interval training under hypoxia on endurance exercise performance in relation to hemodynamic function and exercise economy.

The present study confirmed that our hypoxic training method effectively improved hemodynamic function and exercise economy by decreasing HR and VO_2_ and increasing SV during submaximal cycle ergometer exercise for 30 min versus normoxic training in amateur male swimmers. Hemodynamic function, including exercise economy and VO_2max_, are important factors that determine performance in endurance athletes [35,36,37]. Enhancing the exercise economy means improved adenosine triphosphate (ATP) resynthesis (per 1 mol of O_2_) and reduced ATP levels at a given exercise load [1,10,35]. In addition, the enhancement of exercise economy not only increases the efficiency of oxygen delivery and use capacity and energy availability; it also improves the invigoration of the PNS via activation of β-adrenergic receptors in the cardiac muscles and increases venous return, thereby effectively altering cardiac function [1,10,12]. Our study reported that aerobic continuous and interval training under hypoxia enhanced endurance exercise performance (e.g., VO_2max_ and 400-m time trial record) with improved exercise economy (decreased VO_2_ during submaximal exercise), venous return (decreased HR during submaximal exercise), and pumping ability of the heart (increased SV during submaximal exercise) than in normoxia in amateur male swimmers. These results are important for examining the effect of improving exercise performance through hypoxic training.

ANS function can be quantitatively evaluated using HRV and reflects the interaction between the SNS and the PNS on the sinoatrial nodes, which regulate cardiovascular function [21]. HRV is the most effective and reproducible parameter among those obtained from tests for measuring changes in ANS function [38,39]. The HRV test has been mainly used in the fields of mental health examination and health science because it is highly correlated with mental disorders such as depression, anxiety, chronic fatigue syndrome, and insomnia [21,40]. In recent years, the HRV test has been widely used in the field of sports science to more effectively monitor the effectiveness and conditioning of exercise training [10,23,39]. Therefore, we confirmed the effect of aerobic continuous and interval training under hypoxia on ANS function using HRV tests versus normoxia in amateur male swimmers. The HTG showed a significantly improved RMSSD, TP, and LF/HF ratio, but no change was observed in the NTG.

RMSSD is the square root of the mean squared of successive NN interval differences, and it reflects short-term variability in heart rate and is mainly used to predict HRV in HF bands. RMSSD is an index that reflects the PNS and is interpreted as a physiologically healthy and relaxed state as its value increases [24]. TP is an indicator of the overall regulatory ability of the ANS. In the case of chronic stress or physical disease, TP decreases due to a reduction in the ability to regulate the ANS [41]. HF generally reflects the activity of the vagus nerve branching into the heart, while LF refers to the activity of the sympathetic nerve [40]. Therefore, the LF/HF ratio reflects the overall balance of the ANS, and a higher LF/HF ratio indicates that the SNS is relatively elevated or that the PNS activity is suppressed [10,21]. The present study confirmed that aerobic continuous and interval training under hypoxia can improve ANS function via parasympathetic activation and ANS balance by improving the RMSSD, TP, and LF/HF ratio. These results show that our hypoxic training program can improve HRV, that is, ANS function, to obtain a more efficient training effect, and it indicates that the exercise performance ability can be improved.

Among various hypoxic training methods, LLTH has received the most attention from various athletes because it commonly involves shorter exposure time to hypoxia (approximately three to five sessions per week of 1–3 h), less effort, lower cost, and less time [1,10]. In particular, CHT and IHT are most commonly used to enhance endurance exercise performance [1,4,5,10]. Many previous studies examined the exercise performance of swimmers via this type of hypoxic training. Park et al. [1] reported that high-intensity training (<3 h of hypoxic exposure 3 times per week for 6 weeks) composed of aerobic continuous treadmill and anaerobic interval bicycle exercise and normoxic training (swimming and resistance exercise) are effective at enhancing VO_2max_ and 400-m swimming performance via improved exercise economy in competitive swimmers. Truijens [17] tested the hypothesis that high-intensity hypoxic training improves sea-level performance more than equivalent training in normoxia. As a result, they concluded that five weeks of high-intensity training in a flume improves sea-level swimming performance and VO_2max_ in well-trained swimmers with no additive effect of hypoxic training. Czuba [42] evaluated the efficacy of IHT on anaerobic and aerobic capacity and swimming performance in well-trained swimmers. They reported that high-intensity IHT is an effective training method for improving anaerobic capacity and swimming sprint performance. Park and Lim [4] evaluated the effects of six weeks of hypoxic training composed of warm-up, continuous training, interval training, elastic resistance training, and cool-down on exercise performance in moderately trained competitive swimmers. They proposed that hypoxic training is effective for improving muscular strength and endurance but resulted in unclear changes in VO_2max_, anaerobic power, and 50-m and 400-m swimming performance compared to normoxic training.

Our hypoxic training method composed of aerobic continuous and interval training under hypoxia resulted in a clear enhancement of VO_2max_ and 400-m swimming record with improved hemodynamic function (decreased HR and VO_2_, increased SV) during submaximal exercise and ANS function (increased RMSSD and TP, decreased LF/HF ratio) in amateur male swimmers. Although the subjects of the present study were amateur male swimmers at a different initial level from previous studies of elite swimmers, our hypoxic training appears effective at enhancing endurance exercise performance, improvements that may be associated with improved hemodynamic and ANS function.

## 5. Conclusions

Our study demonstrated that six weeks of aerobic continuous and interval training under hypoxia enhanced endurance exercise performance versus normoxia in amateur male swimmers. In addition, the improvement in exercise performance (e.g., VO_2max_ and 400-m time trial record) observed after six weeks of hypoxic training might be due to the increase in hemodynamic function (e.g., HR, VO_2_, and SVi) during submaximal exercise and ANS function assessed by HRV.

## Figures and Tables

**Table 1:** Subjects’ characteristics.

Variable	NTG (*n* = 10)	HTG (*n* = 10)	*p* Value
Environmental condition (mmHg)	Normoxia (760 mmHg)	Hypoxia (526 mmHg)	-
Age (years)	23.90 ± 3.07	24.00 ± 3.06	0.943
Height (cm)	176.3 ± 5.69	176.36 ± 6.04	0.988
Weight (kg)	69.89 ± 13.40	70.18 ± 10.94	0.958
BMI (kg/m^2^)	23.04 ± 2.88	23.05 ± 1.60	0.992
Percent body fat (%)	23.24 ± 3.88	23.28 ± 3.91	0.982

Values are expressed as the mean ± standard deviation. BMI, body mass index; HTG, hypoxic training group; NTG, normoxic training group.

**Table 2:** Changes in body composition before and after training by group.

Measures	NTG (*n* = 10)			HTG (*n* = 10)			*η*^2^ (*p*) Value		
	Before	After	Mean Change (95% CI)	Before	After	Mean Change (95% CI)	Time	Group	Interaction
Weight (kg)	69.89 ± 13.40	69.30 ± 13.24	−0.60 (−1.43, 0.24)	70.18 ± 10.94	70.01 ± 11.34	−0.17 (−1.08, 0.74)	0.098 (0.179)	0.000 (0.928)	0.032 (0.447)
BMI (kg/m^2^)	23.04 ± 2.88	22.58 ± 2.33	−0.46 (−1.54, 0.59)	23.05 ± 1.60	22.44 ± 1.58	−0.61 (−1.00, −0.22)	0.205 (0.045) †	0.000 (0.945)	0.005 (0.766)
Percent body fat (%)	23.24 ± 3.88	23.18 ± 3.65	−0.06 (−1.15, 1.03)	23.18 ± 3.65	22.57 ± 3.90	−0.71 (−1.87, 0.45)	0.062 (0.290)	0.002 (0.867)	0.045 (0.370)

Values are expressed as mean ± standard deviation. BMI, body mass index; CI, confidence interval; HTG, hypoxic training group; NTG, normoxic training group. Significant interaction or main effect: † *p* < 0.05.

**Table 3:** Changes in hematological parameters before versus after training by group.

Measures	NTG (*n* = 10)			HTG (*n* = 10)			*η*^2^ (*p*) Value		
	Before	After	Mean Change (95% CI)	Before	After	Mean Change (95% CI)	Time	Group	Interaction
RBC (×10^6^/µL)	4.70 ± 0.49	4.57 ± 0.50	−0.14 * (−0.22, −0.05)	5.16 ± 0.44	4.84 ± 0.43	−0.32 * (−0.39, −0.24)	0.820 (0.000) †	0.144 (0.099)	0.417 (0.002) †
Hb (g/dL)	14.79 ± 1.43	14.41 ± 1.46	−0.38 * (−0.60, −0.15)	15.51 ± 1.21	14.64 ± 1.31	−0.87 * (−1.11, −0.63)	0.806 (0.000) †	0.033 (0.813)	0.397 (0.003)†
Hct (%)	44.35 ± 3.41	43.95 ± 3.73	−0.40 (−1.29, 0.49)	46.40 ± 3.19	43.99 ± 3.46	−2.41 * (−3.12, −1.70)	0.642 (0.000) †	0.025 (0.502)	0.479 (0.001)†
EPO (mU/mL)	9.11 ± 2.22	10.16 ± 3.47	2.30 (0.87, 3.72)	10.16 ± 3.47	15.25 ± 2.00	5.09 (2.16, 8.01)	0.593 (0.000) †	0.271 (0.019)†	0.173 (0.068)
MCV (μm^2^)	94.58 ± 3.52	96.54 ± 3.40	1.95 (1.59, 2.31)	90.15 ± 2.90	91.03 ± 2.16	0.88 (−0.73, 2.49)	0.456 (0.001) †	0.444 (0.001)†	0.107 (0.159)
MCH (μg)	31.49 ± 1.17	31.61 ± 1.04	0.12 (−0.11, 0.36)	30.14 ± 1.32	30.29 ± 1.30	0.16 (−0.14, 0.46)	0.135 (0.111)	0.258 (0.022) †	0.003 (0.823)
MCHC (g/dL)	33.30 ± 0.84	32.75 ± 0.63	−0.55 (−0.84, −0.26)	33.42 ± 0.91	33.27 ± 0.96	−0.15 (−0.63, 0.33)	0.310 (0.011) †	0.042 (0.395)	0.128 (0.122)

Values are expressed as means ± standard deviations. CI, confidence interval; EPO, erythropoietin; Hb, hemoglobin; Hct, hematocrit; HTG, hypoxic training group; MCV, mean corpuscular volume; MCH, mean corpuscular hemoglobin; MCHC, mean corpuscular hemoglobin concentration; NTG, normoxic training group; RBC, red blood cells. Significant interaction or main effect: † *p* < 0.05; Significant difference before and after training: * *p* < 0.05.

**Table 4:** Changes in hemodynamic function during submaximal exercise for 30 min before and after training by group.

Measure	NTG (*n* = 10)			HTG (*n* = 10)			*η*^2^ (*p*) Value		
	Before	After	Mean Change (95% CI)	Before	After	Mean Change (95% CI)	Time	Group	Interaction
HR (beats/30 min)	4980.3 ± 420.0	4626.6 ± 210.0	−353.7 (−586.1, −121.3) *	4911.3 ± 218.6	4257.9 ± 163.7	−653.4 (−795.4, −511.4) *	0.795 (0.000) †	0.193 (0.053)	0.256 (0.023) †
VO_2_ (mL/kg/30 min)	1130.4 ± 192.1	1114.2 ± 173.4	−16.22 (−49.92, 17.47)	1115.8 ± 161.6	1053.3 ± 152.6	−62.45 (−91.16, −33.74) *	0.473 (0.001) †	0.014 (0.624)	0.237 (0.030) †
SVi (mL/30 min)	1497.8 ± 98.8	1550.1 ± 100.4	52.32 (8.54, 96.10) *	1514.3 ± 194.0	1712.0 ± 110.6	197.66 (90.10, 305.22) *	0.568 (0.000) †	0.135 (0.111)	0.308 (0.011) †
COi (L/30 min)	240.9 ± 18.5	224.9 ± 20.5	−15.95 (−41.72, 9.83)	251.8 ± 31.2	227.6 ± 34.2	−24.25 (−57.80, 9.30)	0.204 (0.045) †	0.043 (0.382)	0.011 (0.662)

Values are expressed as means ± standard deviations. CI, confidence interval; COi, cardiac output index; HTG, hypoxic training group; HR, heart rate; NTG, normoxic training group; SVi, stroke volume index; VO_2_, oxygen uptake. Significant interaction or main effect: † *p* < 0.05; Significant difference before versus after training: * *p* < 0.05.

**Table 5:** Changes in autonomic nervous system function before versus after training by group.

Measures	NTG (*n* = 10)			HTG (*n* = 10)			*η*^2^ (*p*) Value		
	Before	After	Mean Change (95% CI)	Before	After	Mean Change (95% CI)	Time	Group	Interaction
SDNN (ms)	54.06 ± 6.06	55.11 ± 6.60	1.04 (−4.06, 6.15)	52.91 ± 11.54	59.16 ± 9.83	6.25 (−0.39, 12.1)	0.200 (0.048) †	0.009 (0.688)	0.113 (0.147)
RMSSD (ms)	32.18 ± 7.91	35.50 ± 8.81	3.32 (−0.10, 6.74)	31.18 ± 8.31	41.33 ± 9.60	10.15 (4.37, 15.94) *	0.533 (0.000) †	0.025 (0.509)	0.227 (0.034) †
TP (ms^2^)	8.04 ± 0.22	8.24 ± 0.26	0.20 (−0.05, 0.46)	7.75 ± 0.47	8.47 ± 0.40	0.72 (0.50, 0.94) *	0.685 (0.000) †	0.002 (0.843)	0.405 (0.003) †
LF (ms^2^)	6.64 ± 0.48	6.46 ± 0.36	−0.18 (−0.43, 0.63)	6.62 ± 0.49	6.30 ± 0.59	−0.32 (−0.48, −0.17)	0.463 (0.001) †	0.011 (0.661)	0.062 (0.288)
HF (ms^2^)	6.40 ± 0.38	6.56 ± 0.49	0.17 (−0.13, 0.46)	6.01 ± 0.89	6.69 ± 0.65	0.69 (0.20, 1.17)	0.392 (0.003) †	0.015 (0.611)	0.192 (0.053)
LF/HF ratio	1.04 ± 0.071	0.986 ± 0.048	0.054 (−0.109, 0.001)	1.12 ± 0.178	0.948 ± 0.127	−0.173 (−0.277, −0.069) *	0.516 (0.000) †	0.012 (0.644)	0.226 (0.034) †
Serum cortisol (μg/dL)	14.41 ± 5.23	12.97 ± 3.75	−1.44 (−4.50, 1.62)	14.29 ± 1.63	10.93 ± 2.37	−3.36 (−5.48, −1.24)	0.320 (0.009) †	0.035 (0.430)	0.070 (0.258)

Values are expressed as mean ± standard deviation. CI, confidence interval; HF, high-frequency; HTG, hypoxic training group; LF, low-frequency; NTG, normoxic training group; RMSSD, root mean square of successive differences; SDNN, standard deviation of NN intervals; TP, total power. Significant interaction or main effect: † *p* < 0.05; Significant difference before versus after training: * *p* < 0.05.

## Data Availability

The data presented in this study are available on request from the corresponding author.
